# Worldwide variability in deceased organ donation registries

**DOI:** 10.1111/j.1432-2277.2012.01472.x

**Published:** 2012-04-16

**Authors:** Amanda M Rosenblum, Alvin Ho-Ting Li, Leo Roels, Bryan Stewart, Versha Prakash, Janice Beitel, Kimberly Young, Sam Shemie, Peter Nickerson, Amit X Garg

**Affiliations:** 1 Division of Nephrology, Department of Medicine, London Health Sciences CentreLondon, Ontario, Canada; 2 Donor Action FoundationLinden, Belgium; 3 OneLegacyLos Angeles, CA, USA; 4 Donate Life America Donor Designation Collaborative FacultyRichmond, VA, USA; 5 Trillium Gift of Life NetworkToronto, Ontario, Canada; 6 Organs and Tissues, Canadian Blood ServicesEdmonton, Alberta, Canada; 7 Division of Pediatric Critical Care, Montreal Children's Hospital, McGill University Health CentreMontreal, Quebec, Canada; 8 Department of Internal Medicine, University of ManitobaWinnipeg, Manitoba, Canada; 9 Department of Epidemiology and Biostatistics, University of Western OntarioLondon, Ontario, Canada

**Keywords:** donation, donor identification, donor registry, legal aspects, organ preservation & procurement, organization

## Abstract

The variability in deceased organ donation registries worldwide has received little attention. We considered all operating registries, where individual wishes about organ donation were recorded in a computerized database. We included registries which recorded an individual's decision to be a donor (donor registry), and registries which only recorded an individual's objection (non-donor registry). We collected information on 15 characteristics including history, design, use and number of registrants for 27 registries (68%). Most registries are nationally operated and government-owned. Registrations in five nations expire and require renewal. Some registries provide the option to make specific organ selections in the donation decision. Just over half of donor registries provide legally binding authorization to donation. In all national donor registries, except one, the proportion of adults (15+) registered is modest (<40%). These proportions can be even lower when only affirmative decisions are considered. One nation provides priority status on the transplant waiting list as an incentive to affirmative registration, while another nation makes registering a donation decision mandatory to obtain a driver's license. Registered objections in non-donor registries are rare (<0.5%). The variation in organ donor registries worldwide necessitates public discourse and quality improvement initiatives, to identify and support leading practices in registry use.

## Introduction

Organ donation registries are computerized databases that record member donation wishes. This is so a proper decision can be made on behalf of the deceased when the registry is checked by authorized personnel at the time of death. There are two distinct types of registries, donor and non-donor registries. Donor registries record an individual's decision to be a deceased donor. They are also used to promote organ donor awareness and evaluate public campaigns [1–3]. This is important as transplantation improves survival, quality of life and is cost saving, yet there is an inadequate number of organs available for those in need [4–8]. Non-donor registries record an individual's objection to deceased donation. They are not designed to promote deceased donation, but instead are a legal tool for individuals to express their objection.

Whether donor registries effectively improve transplantation rates remains an open question. Nonetheless their use continues to expand, supported by the American Society of Transplantation and the public in many nations [9–11]. Many characteristics of organ donation registries differ between nations. Highlighting this variation is useful for public discourse and guides discussions about leading practices amongst registry providers. We conducted this review to address this information need.

## Methods

### Definitions

Our use of the term ‘donor’ registry refers to registries that record either affirmative decisions only or both affirmative decisions and objections. A ‘non-donor’ registry refers to registries that only record an objection to donation. The term ‘registries’ refers to both donor and non-donor registries.

In an explicit consent (opt in) system an individual records their decision to become an organ donor in the event of their death. They then become an organ donor if their decision is registered in a donor registry or expressed by family members at the time of death. Donor registries can record legally binding ‘authorization’ or a non-binding expression of ‘intent’ by the deceased to donate. Some donor registries only record affirmative decisions while others record both affirmative decisions and objections.

Non-donor (or objection-only) registries are used only in nations with presumed consent (opt-out) donation legislation [12]. In such a system, an individual opposed to organ donation either registers their decision not to donate in the event of their death, or expresses this decision to their family members. Otherwise, it is understood the individual will become an organ donor. It is important to note that not all nations with presumed consent use non-donor registries. Rather, some nations use registries that record both objections and affirmative decisions, and some only record affirmative decisions.

### Data of interest

We considered all nations with active deceased organ donation programs. We collected information relevant to the design and use of each registry including: implementation date, ownership, operation level (national or regional), minimum eligible age, expiration period of registered decision (if applicable), registration options (registration choices, ability to specify organs to include and/or exclude), available methods of registration, priority status on transplant wait list, mandated choice, accessing the registry at the time of death (use in procurement process, authorized person, method of access), legal status of registered choices and registrant values (described according to registration choice). To calculate the registration proportions we defined the adult population as the total population 15 years of age and older using values from the Central Intelligence Agency World Factbook estimated for July 2011 [13]. Fifteen and older was used to create a base denominator among nations, in order to facilitate comparisons. However since not all nations use 15 as a minimum age requirement, exact proportions of the population registered will vary.

State and provincial registries operate in the USA and Canada respectively. When making generalizations about registry characteristics across these two nations, we described what the majority of states and provinces did. The only exception was for expirations; several states and provinces have registrations that must be renewed, and therefore warranted attention. For the total number of registrants, we used unweighted averages for both the USA and Canada. When providing registration information at the regional level, for American states the adult population was defined as the total population 18 years of age and older using values from the US Census Bureau [14]. For Canadian provinces the adult population was defined as the total population 15 years of age and older using values from Statistics Canada [15]. Again, exact proportions will vary for states and provinces where 18 and 15 are not used as a minimum age requirement.

### Data collection

Data was collected from November 2010 until June 2011. A single author (AMR) first determined if there was an active registry by searching published literature and conducting Internet searches of ministries of health, nephrology and transplantation foundations’ websites. Relevant data was abstracted by this same author (AMR) into Excel 2007 (Microsoft, Redmond, Washington, DC, USA). We sent our data to registry personnel for review and to supplement any missing fields. A second independent reviewer (AL) then reviewed all the data for accuracy including responses from nation representatives.

## Results

### Registries included in review

The Global Observatory on Donation and Transplantation identifies 60 nations as having active deceased organ donation programs [16]. Lebanon is not identified as active, but nation representatives confirmed they have a deceased donation program and an active donor registry. Therefore 61 nations were considered eligible for review. We determined that 20 nations do not have active deceased donation registries, and all exclusions were confirmed by nation representatives. We excluded one nation from our study because of political unrest (Tunisia, non-donor registry). Of the 40 nations left for consideration, registries operating in 27 nations (68%) were included in the review. For the remaining 13 nations, complete information was either unavailable or nation representatives were unresponsive. This precluded knowledge of whether the 13 nations have active registries. A list of all 61 nations subdivided into included, excluded and unresponsive categories is presented in Appendix S1.

### Data tables

Characteristics of the registries included in this review are presented in Table 1, separated into registries in nations with explicit consent for deceased organ donation (Table 1a), registries in nations with presumed consent for deceased organ donation where the registry included affirmative registration (Table 1b), and non-donor objection only registries in nations with presumed consent (Table 1c). Similar regional information for individual American states and Canadian provinces are presented Appendices S2 and S3, respectively.

**Table 1:** (a) Characteristics of donor registries operating in nations with explicit consent for deceased organ donation. (b) Characteristics of donor registries operating in nations with presumed consent for deceased organ donation. These registries include affirmative registration in support of deceased organ donation. (c) Characteristics of non-donor registries in nations with presumed consent for deceased organ donation

(a)											

					Organ specification		Registration modalities				
							
Nation	Implementation date	Operation level	Registration choices	Minimum age	Can registrants specify which organs to donate?	Are specified organs to be included or excluded from donation?	Online	Paper	Telephone	In person	Additional details
Australia	2000	National†	Yes & no	16	Yes	Include	Yes	Yes	Yes	No	Individuals 16–17 can register their intent, but must be 18+ to register legal authorization
Canada	1995–2007‡	Regional	Yes & no‡	None-18‡	Yes‡	Include & exclude‡	Yes‡	Yes‡	Yes‡	Yes‡	Described in Appendix S3
Denmark	1990	National	Other	18	Yes	Include	Yes	Yes	No	No	Registration choices include ‘yes’, ‘no’ and ‘unsure’. Individuals can also add ‘with next of kin approval’ to their registration
Iran	2000	Regional	Yes only	18	Yes	Include & exclude	Yes	Yes	No	Yes	In person registration is available at the organ procurement units
Israel	1978	National	Yes only	17	Yes	Exclude	Yes	Yes	Yes	Yes	Individuals have the option of ‘yes’ and ‘yes with religious permission’. In person registration is available at some coffee shops. A pre-paid postcard is provided for mailed registrations
Kuwait	1988	National	Yes only	21	No^§^	n/a	Yes	Yes	No	Yes	Registry became computerized in 1996. In person registration is available at the Kuwait Transplant Society or transplant centre
Lebanon	2007	National	Yes only	15	Yes	Include	Yes	No	Yes	Yes	Parental authorization is mandatory by law. In person registration is available at the National Organization for Organ and Tissues Donation and Transplantation, Eye Bank Center in Quarantine Hospital, and the Lebanese Order of Physicians
Malaysia	1997	National	Yes only	18	Yes	Include	Yes	Yes	No	No	Registrants below the age of majority must have parental authorization. Forms are available online, in post offices and hospitals, by telephone request and given out during public campaigns
Netherlands	1998	National	Other	12	Yes	Exclude	Yes	Yes	No	No	Four registration options are ‘yes’, ‘no’, ‘next of kin will decide’ or ‘a specified person will decide’
New Zealand	1980s	National	Yes & no	15	No	n/a	No	No	No	Yes	The organ donor question on a driver's license form is compulsory. Decisions are renewed every 10 years with driver's license renewal. In person registration is available at Land Transport New Zealand. Registrants can also update their donor information by telephone
UK	1994	National	Yes only	None	Yes	Include	Yes	Yes	Yes	Yes	Authorization is sought from the next of kin for those donors under 18. In person registration is available through registering for a driver's license, applying for a Boots Advantage card, through a physician, through registering for a European health Insurance Card, and through text message
USA	1980s–2010¶	Regional	Yes only	None-18¶	Yes¶	Include & exclude¶	Yes¶	Yes¶	Yes¶	Yes	Described in Appendix S2

(b)											
Argentina	2006	National	Yes & no	18	Yes	Include	No	No	No	Yes	In person registration is available at organ procurement organizations, the civil registry, and the federal police. Objections can also be made in post offices via telegram, free of charge
Belgium	1987	National	Yes & no	None	No	n/a	No	Yes	No	Yes	As there is no minimum age restriction, parents may register their children and minors ‘capable to express their will’ can register. Once a registrant turns 18, they are invited to confirm the registered will made by their parents. In person registration is available at municipal town halls
Colombia	2006	National	Yes only	18	No	n/a	Yes	No	No	Yes	In person registration is available at the National Health Headquarters
Italy	2000	National	Yes & no	18	No	n/a	No	No	No	Yes	In person registration is available at local health authorities
Lithuania	2000	National	Yes & no	18	Yes	Include & exclude	No	Yes	No	Yes	In person registration is available at any health care institution and through a family physician
Slovenia	2004	National	Yes only	15	No	n/a	No	No	No	Yes	All individuals with health insurance are considered ‘undefined’ until they register ‘yes’. Registration is only available by the completion of an official form in person in the presence of a person authorized by Slovenija-transplant at designated areas (certain health institutions, pharmacies, Red Cross, Blood Transfusion Institute)
Sweden	1996	National	Yes & no	None	Yes	Exclude	Yes	Yes	No	No	When registered minors turn 18 they receive a letter from the registry with their donor information so that they can make any changes if necessary

(c)										
Austria	1995	National	16	Yes	Include & exclude	No	Yes	No	Yes	In person registration is available at the health department. Individuals also have the option of registering via email
Croatia	2005	National	18	No	n/a	No	No	No	Yes	Registration is only available through a family physician
Czech Republic	2004	National	None	Yes	Include	No	Yes	No	Yes	In person registration is available in hospital
France	1997	National	13	No	n/a	No	Yes	No	No	
Hungary	1999	National	None	No	n/a	No	Yes	No	Yes	While there is no minimum age restriction, parents of registrants who are under 18 may protest. In person registration is available at government offices or through a family physician
Poland	1996	National	16	No	n/a	No	Yes	No	Yes	In person registration is available through Poltransplant
Portugal	1993	National	None	Yes	Exclude	No	No	No	Yes	In person registration is available through any health centre
Slovakia	1997	National	18	No	n/a	No	Yes	No	Yes	Children under the age of 18 may register with parental authorization

† New South Wales still operates a separate registry through their road transport authority.

‡ Varies by province, see Appendix S3.

§ There is a Notes section where individuals could write organs to include or exclude.

¶ Varies by state, see Appendix S2.

Details on how each registry is accessed and used at the time of death is presented in Table 2, separated into registries in nations with affirmative registration (Table 2a), and non-donor objection-only registries (Table 2b). Similar regional information for individual American states and Canadian provinces are presented in Appendices S4 and S5, respectively.

**Table 2:** (a) How the donor registry is accessed and utilized at the time of death in nations with affirmative registration. (b) How the non-donor registry is accessed and used at the time of death in nations with objection-only registries

(a)			

Nation	Intent or legal authorization	How the is registry accessed by staff	Personnel authorized to access the registry at the time of death
Argentina	Legal authorization	Computer	Different levels of personnel in the procurement system are provided with a password by INCUCAI (national transplant organization)
Australia	Intent & legal authorization	Computer	Medical professionals that include transplant coordinators, eye and tissue bank staff, and intensive care specialists
Belgium	Legal authorization	Computer	Transplant coordinators authorized with a password
Canada	Intent & legal authorization†	Computer or telephone†	Varies by province, see Appendix S5. A mix of procurement staff, government staff, transplant organization staff, nurses, physicians, and tissue bank staff
Colombia‡	Intent	Computer	National Network of Donors staff and National Health Institute staff
Denmark	Legal authorization	Computer or telephone	Health care professionals only
Iran	Intent	Computer	Manager of deceased donation organ procurement unit
Israel	Intent	Computer	Ministry of Health's National Transplant Center staff
Italy	Intent	Computer	National, regional and inter-regional transplant centers
Kuwait	Legal authorization	Telephone	Transplant center staff and hospital ICU staff
Lebanon	Intent	Telephone	Transplant coordinators
Lithuania	Legal authorization	Computer	Lithuanian National Transplantation Bureau (NTB) staff
Malaysia	Intent	Telephone	National Transplant Resource Center staff
Netherlands	Legal authorization	Computer or telephone	Protocol varies by hospital but is a specific staff member or physician
New Zealand§	Intent	Telephone	Transplant and tissue coordinators
Slovenia	Legal authorization	Computer	Transplant coordinators and medical co-workers
Sweden	Legal authorization	Computer or telephone	Transplant coordinators, tissue coordinators, forensic medicine personnel
UK	Legal authorization	Computer & telephone	NHS Blood and Transplant employees working in the organ exchange part of the organization
USA	Legal authorization	Computer or telephone¶	Varies by state, see Appendix S4. A mix of organ procurement staff, tissue bank staff, eye bank staff, and government staff

(b)		

Nation	How is the registry accessed by staff	Personnel authorized to access the registry at the time of death
Austria	Telephone	Medical personnel with special code word
Croatia	Computer	National and hospital transplant coordinators
Czech Republic	Computer	Czech Transplantations Coordinating Center (KST) staff and local coordinators
France	Fax	Hospital personnel involved in the organ procurement process
Hungary	Telephone or fax	Designated medical staff
Poland	Computer	Transplant coordinators working at Polish Transplant Coordinating Center (Poltransplant)
Portugal	Computer	Authorized organ donation bureaus
Slovakia	Telephone or fax	Authorized Slovak Centre for Organ Transplantation (SCOT) staff

† Varies by province, see Appendix S5.

‡ Registration is symbolic and the registry is not checked at the actual time of procurement.

§ Registration is only checked upon the family's request.

¶ Varies by state, see Appendix S4.

Note (b): All registrations in the non-donor registries are considered legally binding objections to donation.

Nation registry values are presented in Table 3 and for individual American states and in Appendices S6 and S7, respectively.

**Table 3:** (a) Number and proportions of registrants for donor registries (all nations that include affirmative registration). (b) Number and proportions of registrants for non-donor registries

(a)						

Nation	Total adult population	Total registrants	Total affirmative registrants	Total objecting registrants	Proportion of the population registered (%)	Proportion of population registered as affirmative (%)
Argentina	31 160 216	2 903 747	2 405 706	498 041	9.32	7.72
Australia	17 783 403	5 700 332	5 682 688†	17 644	32.05	31.96
Belgium	8 772 872	278 671	89 864	188 807	3.18	1.02
Canada‡	28 753 718	4 319 804	3 614 396	250 333	15.02	12.57
Colombia	32 783 823	119 738	119 738	–	0.37	0.37
Denmark	4 556 628	690 000	650 000	40 000	15.14	15.14
Iran	59 119 436	600 000	600 000	–	1.01	1.01
Israel	5 410 490	560 000	560 000	–	10.35	10.35
Italy	52 596 485	1 226 731	1 213 576	13 155	2.33	2.31
Kuwait	1 925 956	4 373	4 373	–	0.23	0.23
Lebanon	3 190 188	3 000	3 000	–	0.09	0.09
Lithuania	3 047 642	14 204	14 157	47	0.47	0.46
Malaysia	20 224 939	149 315	149 315	–	0.74	0.74
Netherlands	13 983 016	5 558 527§	3 256 219	1 605 909	39.75	23.29
New Zealand	3 415 116	3 700 083	1 732 958	1 967 125	100.00¶	50.74
Slovenia	1 732 080	2 243	2 243	–	0.13	0.13
Sweden	7 689 064	1 500 000	960 000	540 000	19.51	12.49
UK	51 851 545	17 400 213	17 400 213	–	33.56	33.56
USAΦ	250 272 403	96 417 971	96 417 971	–	38.53	38.53

(b)			

Nation	Total adult population	Total objecting registrants	Proportion of the population registered (%)
Austria	7 066 861	21 000	0.30
Croatia	3 806 750	1 600	0.04
Czech Republic	8 814 534	941	0.01
France	53 058 716	81 600	0.15
Hungary	8 489 629	723‡	0.01
Poland	32 790 675	25 647	0.08
Portugal	9 017 136	38 246	0.42
Slovakia	4 622 620	265	0.01

Notes (a): Adult population is defined as those 15 years of age and older, and was calculated from CIA World Factbook. Exact proportions will vary slightly for nations with no minimum age and for those with age minimums higher than 15. Please see Table 2 for each nation's minimum age requirements.

†Australia's affirmative registrations include 1 416 622 legal authorizations and 4 266 066 intent registrations.

‡Described by province in Appendix S7.

§Netherlands also have the options “Next-of-kin will decide” (594 698 registrants) and “A specified person will decide” (101 701 registrants).

¶The actual value is 108.34. Possible reasons for the discrepancy between total registrants and 15+ population (15 is the minimum age to register) include that many New Zealanders live abroad and that the driver's license renewal period is every 10 years. In order to receive a driver's license one must record their donation decision.

ΦDescribed by state in Appendix S6.

Notes (b): Adult population is defined as those 15 years of age and older, and was calculated from CIA World Factbook. Exact proportions will vary slightly for nations with no minimum age and for those with age minimums higher than 15. Please see Table 2 for each nation's minimum age requirements.

‡Due to regulation, Hungary is unable to give a current figure. This figure comes from a study by Gabel [22].

### Implementation date

Israel has the oldest registry, which was implemented in 1978. Registries have become more common in the last two decades, with a number of new registries in the last five years (Lebanon, several American states (including Florida, New Hampshire and South Carolina), and two Canadian provinces (New Brunswick and Quebec)).

### Ownership and operation level

Nationally operated and government-owned registries are the most common (89% and 81% of nations, respectively). The three nations with regional registries are the USA, Canada and Iran. The USA and Canada have regional registries because deceased donation legislation falls under state/provincial legal jurisdiction. Also organ donation is linked to renewable government services managed at the state or province level, such as the department of motor vehicles and health insurance. Iran originally intended to have a national registry but changed to a regional system run by the 10 organ procurement organizations. This was done to provide more options for Iran's 31 provinces. Three nations (Australia, France and Lebanon) switched from having regional registries to a single national registry.

### Minimum age requirements

Twenty-two nations (81%) have a minimum age requirement in order to register. In regions that do not, such as the UK, Sweden and about half of the American states, registrations can be made by individuals considered ‘minors’ if parental authorization is given at the time of registration and/or parents are responsible for making the final decision at the time of procurement.

### Expirations

Five nations (19%) have registrations that expire. This often occurs in settings where the registrations are made through a driver's license or state identification card, with reaffirmation required when the license/card expires. In Belgium and Slovakia, registrations for individuals under the age of 18 that are made by a parent or guardian expire once the registrant turns 18.

### Registration choices and organ specification

Eight nations have registries that record both ‘yes’ and ‘no’ responses (an affirmative choice or objection to donation, respectively), nine nations record only ‘yes’ responses, and eight nations record only ‘no’ responses. Two nations (Denmark and the Netherlands) are classified as ‘other’ because they offer more options than ‘yes’ or ‘no’. Registrants in Denmark can choose ‘yes’, ‘no’ or ‘unsure’. They may also add the condition ‘with next of kin approval’ to their registration. The Netherlands offers the choice of ‘yes’, ‘no’, ‘next of kin will decide’ or ‘a named individual will decide’. While Israel records affirmative choices only, individuals have the option of checking ‘yes’ or ‘yes with religious permission’.

Seventeen nations (63%) allow registrants to specify which organs to include or exclude from donation. Five of these nations (Austria, Czech Republic, France, Portugal and Slovakia) are non-donor registries, so registrants choose which organs to include or exclude from their donation objection. The ability to specify organs to include or exclude in the donor designation is more common in nations with explicit consent (83%) than in nations with presumed consent (40%).

### Methods of registration

Of the four most common registration modalities, in-person registration is the most frequently available (71%), followed by paper (mail/fax, 61%), online (50%), and telephone (29%). There are also some uncommon methods, including email (Austria) and select coffee shops (Israel). The UK has the most opportunities for registration, including applying online, and through telephone, driver's license, pharmacy advantage card, a physician, registration for a European Health Insurance Card and text message. None of the non-donor registries have online or telephone registration.

### Priority status

Priority status is the practice of providing preference to individuals on the transplant waiting list who have registered to be deceased donors over those who have not. Israel is the only nation included in this study that has implemented this policy (in 2010). Priority is also extended to registrants’ first degree relatives and to non-directed living donors [17]. A similar policy is also in place in Singapore, where the registrants gain priority if they agree to be deceased donors, and lose priority if they opt out of donation [18].

### Mandatory choice

Mandatory or mandated choice is an approach in which individuals are required to register their donation choice. New Zealand is also the only nation where indicating one's donation decision is compulsory in order to obtain a driver's license.

### Accessing registry information at the time of death

Health care providers for all nations with registries included in this review have a discussion with the next-of-kin about deceased donation as part of the organ procurement process. A registration can then be printed or verbally communicated by authorized personnel to the next-of-kin. All but two nations (Colombia and New Zealand) indicated that they always consult their registry once a potential donor is referred and prior to discussion with the next-of-kin. Colombia's registry is more symbolic in nature, and is never used in the actual procurement process, while the New Zealand registry is only consulted if the next-of-kin requests that a search be made.

Registries are most commonly accessed through a computer (19 nations, 70%), and some systems offer additional telephone access. Computer access is less common in non-donor registries (50% compared to 79% of registries that include affirmative registrations).

Individuals authorized to consult the registry vary by nation but mostly include individuals typically involved in the procurement process (e.g. transplant coordinators, national transplantation organization staff). Access is usually restricted to protect the privacy of registrants.

### Legal status of registered choices

While proof of registration may be used in the procurement consent process, not all registrations are considered legally binding. Registrations that fulfill the legal requirements for authorization and/or objection to deceased donation are valid legal documents and provide legal authorization for procurement to proceed. However, some nations still prefer to consider registrations as an indication of the deceased's intentions that are used in discussions with next-of-kin. Of the 19 donor registries that record affirmative registrations, 12 (63%) record decisions that are legally binding. Exceptions include two Canadian provinces (New Brunswick and Yukon) that record the intent to donate. Australia's donor registry was originally an ‘intention’ registry, but later changed to be legally binding. Australia records both legally binding authorizations and intent registrations. All registrations in the eight non-donor registries are considered legally binding objections to donation.

### Proportion of adults (15+) registered

Of the 19 donor registries, New Zealand (with mandatory choice) has the most registrants with 100% of their adult population registered (51% affirmative registrations). The Netherlands has the second highest proportion of registrations (40% of the adult population registered, 23% affirmative registrations). None of the non-donor registries have proportions registered higher than 0.5%.

When values in regional registries are considered, there are dramatic differences across American states and across Canadian provinces. In the USA only affirmative registrations are recorded. In the USA, the state of Alaska has the highest proportion registered (78%) while Vermont has a strikingly low value (0.3%). The later has been attributed to registration not being affiliated with its department of motor vehicles. In Canada, the province of New Brunswick has the most registrations (78% of adults), while Nova Scotia has the highest affirmative registrations (65%).

## Discussion

To our knowledge, this is the most comprehensive and current global review of active donor and non-donor registries worldwide. We examined multiple characteristics covering the history, design, and use of registries, as well as the number of registrants. The results highlight the considerable variability in deceased donor registries worldwide. Most but not all registries are nationally operated and government owned. There is usually a specific minimum age requirement in order to register. Some registries provide registrants with the option to select specific organs to include and/or exclude from their donation decision. Most registries are consulted by health professionals involved in organ procurement through a computer after a donor referral and before discussion with the next-of-kin. Just over half of the donor registries are considered legally binding authorization to donation. In all national donor registries, except New Zealand, the proportion of adults (15+) registered (either affirmative decisions or objections) is modest (<40%), and is often even lower when only affirmative decisions are considered. Registered objections in non-donor registries are rare (<0.5%).

There was also considerable variation amongst state and provincial donor registries in the USA and Canada, respectively. While this may be done for good reason, present inconsistencies may contribute to the large number of Americans and Canadians who indicate that they are confused about how to become an organ donor [11,19].

When contrasting national registries there a very important distinction between donor and non-donor registries. Both are primarily used to inform the procurement process and ensure a proper decision is made on behalf of the deceased. Donor registries are most often used in nations with explicit consent for deceased organ donation. These registries are used in the promotion of deceased donation, and can be used to target, measure and evaluate public awareness campaigns in support of organ donation. Non-donor registries in contrast are used in some presumed consent nations as a legal means for individuals to object to being a donor. They are not part of a strategy to improve deceased donation, and low proportions of their adult population registered (currently <0.5% in all nations) may be viewed positively by proponents of organ donation. Interesting, some registries operating in presumed consent nations record both objections and affirmative decisions. These types of registries were considered as donor registries for this review. However in some of these nations (such as Belgium), registries were originally conceived as objection to donation registries, and only later expanded to also record affirmative decisions.

New Zealand is the only nation that was studied that makes registration compulsory in order to obtain a driver's license. The high proportion of adult registrants (essentially 100%) suggests that this style of mandated choice helps overcome apathy to register a donation decision. However, some individuals may be unprepared when making their decision, making an uninformed or inaccurate choice. In New Zealand only half of the registrations are affirmative. In comparison, in the USA where all donor registries are affirmative only, some American states have proportions of adult registrants that exceed 70%.

The strengths of our review include both the number of registries that were studied and the range of registry characteristics that were considered. The review extends previous studies which are smaller in scope and typically limited to European nations [20–22]. These results help inform the development of new registries, and allow nations with active registries to frame their programs in a global context. However, our study was limited by the poor availability of published data on the individual registries. This prevented us from collecting information on 13 nations and caused a large reliance for information from nation representatives. We also defined the adult population to be 15 years of age and older to create a base denominator among nations and to facilitate comparisons. However since not all nations use 15 as a minimum age requirement, exact proportions will vary. Finally, our study was not designed to confirm or refute whether donor registries effectively increase deceased donation rates.

Future studies are needed to investigate whether donor registries successfully improve the number of deceased donors. Direct comparisons need to be made between rates of deceased donation in nations with and without registries. There also needs to be an evaluation of individual registry design elements, so that specific recommendations for effective registry design can be made. This could be accomplished through studies that measure how much each design element (e.g. affirmative-only registry versus an affirmative and objecting registry) contributes to improved registration values and improved donation rates. Finally the influence of population preferences on the decision to register should be further investigated, as what constitutes effective design may vary by a nation's ideals and principles.

In conclusion, we show registries are common around the world and that they vary in their objectives, design and use. This information can now be used to prompt public discourse and quality improvement initiatives amongst registry providers, to identify and support leading practices in registry use.

## Supporting information

**Appendix S1.** List of Nations with Deceased Donation Programs.

**Appendix S2.** Characteristics of Donor Registries Operating in the United States.

**Appendix S3.** Characteristics of Donor Registries Operating in Canadian Provinces.

**Appendix S4.** How the Donor Registry is Accessed and Utilized at the Time of Death in the United States.

**Appendix S5.** How the Donor Registry is Accessed and Utilized at the Time of Death in Canada.

**Appendix S6.** Number and Proportions of Registrants for United States.

**Appendix S7.** Number and Proportions for Registrants Canada.
