# Supplementary material for: Worldwide variability in deceased organ donation registries
Source: Transpl Int. 2012 Apr 16;25(8):801–11. doi: 10.1111/j.1432-2277.2012.01472.x (PMC3440579; doi:10.1111/j.1432-2277.2012.01472.x)
Supplement: Supplementary file 1 [file tri0025-0801-SD1.doc]

**SUPPLEMENTAL DIGITAL CONTENT**

Appendix 1 List of Nations with Deceased Donation Programs

Appendix 2 Characteristics of Donor Registries Operating in the United States

Appendix 3 Characteristics of Donor Registries Operating in Canadian Provinces

Appendix 4 How the Donor Registry is Accessed and Utilized at the Time of Death in the United States

Appendix 5 How the Donor Registry is Accessed and Utilized at the Time of Death in Canada

Appendix 6 Number and Proportions of Registrants for United States

Appendix 7 Number and Proportions for Registrants Canada

| **Appendix 1 | List of Nations with Deceased Donation Programs** | |
| --- | --- |
| ***Nations Included in Review*** |  |
| Argentina | Kuwait |
| Australia | Lebanon |
| Austria | Lithuania |
| Belgium | Malaysia |
| Canada | Netherlands |
| Colombia | New Zealand |
| Croatia | Poland |
| Czech Republic | Portugal |
| Denmark | Slovakia |
| France | Slovenia |
| Hungary | Sweden |
| Iran | United Kingdom |
| Israel | United States |
| Italy |  |
| ***Nations Excluded from Review*** |  |
| Bolivia | Mexico |
| Brazil | Myanmar |
| Cyprus | Norway |
| Dominican Republic | Romania |
| Estonia | Saudi Arabia |
| Finland | South Africa |
| Germany | Spain |
| Iceland | Switzerland |
| Ireland | Tunisia^†^ |
| Japan | Turkey |
| Luxembourg |  |
| ***Nations Unresponsive to Review*** |  |
| Bulgaria | Paraguay |
| Chile | Republic of Korea |
| Cuba | Singapore |
| Greece | Thailand |
| Guatemala | Uruguay |
| Latvia | Venezuela |
| Panama |  |

**^†^ Tunisia has an active objection-only registry but was excluded because of political unrest at the time of data collection.**

| **Appendix 2| Characteristics of Donor Registries Operating in the United States** | | | | | | | | | | |
| --- | --- | --- | --- | --- | --- | --- | --- | --- | --- | --- |
| **State** | **Implementation Date** | **Minimum Age** | **Organ Specification** | |  | **Registration Modalities** | | | | **Additional Details** |
|  |  |  | **Can registrants specify which organs to donate?** | **Are specified organs to be included or excluded from donation?** |  | **Online** | **Paper** | **Telephone** | **DMV** |  |
| Alabama | 1997 | 18 | Yes^†^ | Include |  | Yes | Yes | Yes | Yes | In person registration is available at designated kiosks. |
| Alaska | 2004 | None | Yes^†^ | Include |  | Yes | Yes | No | Yes | Registrants must be 14 years of age to sign up through the DMV. In person registration is available at designated kiosks. |
| Arizona | 2003 | 15½ | Yes^†^ | Include & Exclude |  | Yes | Yes | No | Yes | In person registration is available at designated kiosks. |
| Arkansas | 1997 | None | No | n/a |  | Yes | No | No | Yes | In person registration is available at the Department of Motor Vehicles. Registrations made through a driver’s license or state identification card must be reaffirmed at renewal every four years. |
| California | 2005 | 13 | Yes^†^ | Exclude |  | Yes | Yes | No | Yes | Registrants must be 16 years of age to register through the DMV. In person registration is available through mobile laptops at various donation awareness events. |
| Colorado | 2001 | None | Yes^†^ | Include & Exclude |  | Yes | Yes | Yes | Yes | While there is no age minimum to register, parental/guardian authorization is still sought at the time of procurement for those less than 18 years of age. Registrants must be 16 years of age to register through the DMV. |
| Connecticut | 2005 | 16 | Yes^†^ | Exclude |  | Yes | Yes | No | Yes | Registrants must be 16 years of age to register through the DMV. Registrants must be 18 years of age to register online. |
| Delaware | 1998 | None | No | n/a |  | Yes | No | No | Yes | While there is no age minimum to register, parental/guardian authorization is still sought at the time of procurement for those less than 18 years of age. In person registration is available through mobile laptops at various donation awareness events. Registrations made through a driver’s license or state identification card must be reaffirmed at renewal every five years. |
| District of Columbia | 2006 | 13 | Yes^†^ | Exclude |  | Yes | Yes | No | Yes |  |
| Florida | 2009 | None | Yes^†^ | Exclude |  | Yes | Yes | No | Yes |  |
| Georgia | 1996 | None | Yes | Exclude |  | Yes | Yes | No | Yes | The registry was re-launched in 2009. While there is no age minimum to register, parental/guardian authorization is still sought at the time of procurement for those less than 18 years of age. Registrants must be 15 years of age to register through the DMV. In person registration is available through mobile laptops at various donation awareness events. |
| Hawaii | 1991 | None | Yes | Include |  | Yes | Yes | Yes | Yes | While there is no age minimum to register, parental/guardian authorization is still sought at the time of procurement for those less than 18 years of age. |
| Idaho | 2003 | None | Yes^†^ | Include & Exclude |  | Yes | Yes | Yes | Yes | While there is no age minimum to register, parental/guardian authorization is still sought at the time of procurement for those less than 18 years of age. |
| Illinois | 1992 | 18 | No | n/a |  | Yes | Yes | Yes | Yes | The registry became a ‘legally-binding authorization’ registry in 2006. |
| Indiana | 2001 | None | No | n/a |  | Yes | Yes | No | Yes | Registrants must be 16 years of age to register through the DMV. In person registration is available through mobile laptops at various donation awareness events. |
| Iowa | 2007 | None | Yes | Include |  | Yes | Yes | Yes | Yes | Any parent or guardian may add a child 13 years of age or younger to the registry. Registrants who are 14-17 years of age may register with the written permission from a parent or guardian. Registrants 18 years of age and above may register without parental permission. While there is no age minimum to register, parental/guardian authorization is still sought at the time of procurement for those less than 18 years of age. |
| Kansas | 2002 | None | Yes^†^ | Include |  | Yes | No | No | Yes | Registrants must be 16 years of age to register through the DMV. |
| Kentucky | 2006 | None | No | n/a |  | Yes | Yes | No | Yes | While there is no age minimum to register, parental/guardian authorization is still sought at the time of procurement for those less than 18 years of age. In person registration is available through mobile laptops at various donation awareness events. |
| Louisiana | 1997 | None | No | n/a |  | Yes | Yes | Yes | Yes | In person registration is available at designated kiosks. |
| Maine | 2007 | 16 | Yes^†^ | Exclude |  | Yes | No | No | Yes | Registrants must be 18 years of age for online registration. |
| Maryland | 1991 | 13 | Yes^†^ | Include & Exclude |  | Yes | Yes | No | Yes | The registry was re-launched in 2008. Registrants must be 16 years of age to register through the DMV. Registrations made through a driver’s license or state identification card must be reaffirmed at renewal every five years. |
| Massachusetts | 2006 | 16 | Yes^†^ | Exclude |  | Yes | No | No | Yes | Registrants must be 16 years of age to register through the DMV. Registrants must be 18 years of age for online registration. Parental/guardian authorization is still sought at the time of procurement for those less than 18 years of age. Registrations made through a driver’s license or state identification card must be reaffirmed at renewal every five years. |
| Michigan | 1994 | None | No | n/a |  | Yes | Yes | No | Yes |  |
| Minnesota | 2003 | None | No | n/a |  | Yes | Yes | Yes | Yes | Registrants must be 15 years of age to register through the DMV. In person registration is available through mobile laptops at various donation awareness events. |
| Mississippi | 2008 | 18 | Yes^†^ | Include & Exclude |  | Yes | Yes | No | Yes |  |
| Missouri | 1996 | None | Yes^†^ | Exclude |  | Yes | Yes | No | Yes |  |
| Montana | 2004 | 15 | Yes^†^ | Include |  | Yes | Yes | No | Yes |  |
| Nebraska | 2003 | 16 | Yes^†^ | Exclude |  | Yes | Yes | Yes | Yes | In person registration is available through mobile laptops at various donation awareness events. Registrations made through a driver’s license or state identification card must be reaffirmed at renewal. |
| Nevada | 2001 | 16 | Yes | Include |  | Yes | Yes | No | Yes | Parental/guardian authorization is still sought at the time of procurement for those less than 18 years of age. While registration does not expire, registrations must be affirmed while renewing a driver’s license or state identification card at the DMV. |
| New Hampshire | 2009 | 16 | Yes^†^ | Exclude |  | Yes | No | No | Yes | Registrants must be 18 years of age for online registration. |
| New Jersey | 1998 | 18 | No | n/a |  | Yes | Yes | No | Yes | The registry was re-launched in 2009. Registrations made through a driver’s license or state identification card must be reaffirmed at renewal. |
| New Mexico | 1980s | 15 | Yes^†^ | Exclude |  | Yes | Yes | No | Yes | Registrants under the age of 16 require parental or guardian authorization to register. |
| New York | 2008^‡^ | 18 | Yes^†^ | Include |  | Yes | Yes | Yes | Yes | In 1999 there was the Life Pass It on Registry, an intent to donate registry. In 2006 a new law created the New York State Donate Life Registry, a legally-binding authorization registry. These two registries are separate and so registrants in the Life Pass It On Registry must re-enroll in the new registry if they want to record legally-binding authorization to donation. In person registration is available at the state’s organ procurement organizations and tissue banks. Individuals can also register through their voter registration form, although organ specifications cannot be made on it. |
| North Carolina | 2005 | 13 | Yes^†^ | Include |  | Yes | Yes | No | Yes | Registrants must be 16 years of age to register through the DMV. For online registration they must be 18 years of age. Parental/guardian authorization is still sought at the time of procurement for those less than 18 years of age. In person registration is available through mobile laptops at various donation awareness events. While registration does not expire, registrants have the option of reaffirming or removing the donor designation when renewing a driver’s license or state identification card. |
| North Dakota | 2003 | 14 | No | n/a |  | Yes | No | No | Yes |  |
| Ohio | 2002 | 15½ | Yes | Include |  | Yes | Yes | No | Yes | Registrations made through a driver’s license or state identification card must be reaffirmed at renewal every four years. |
| Oklahoma | 2004 | None | Yes^†^ | Exclude |  | Yes | Yes | No | Yes |  |
| Oregon | 2007 | 13 | Yes^†^ | Exclude |  | Yes | Yes | No | Yes | Registrants must be 15 years of age to register through the DMV. In person registration is available through mobile laptops at various donation awareness events. In person registration is available through mobile laptops at various donation awareness events. |
| Pennsylvania | 1994 | 16 | No | n/a |  | Yes | No | No | Yes | Registrants 16-18 years of age are considered minors and cannot complete a full registration online. A form can be printed off and taken to the DMV with parental/guardian signed authorization. A minor can apply in person at the DMV with signed parental/guardian authorization. In person registration is available through mobile laptops at various donation awareness events. Registrations made through a driver’s license or state identification card must be reaffirmed at renewal every four years. |
| Rhode Island | 2005 | 16 | Yes^†^ | Exclude |  | Yes | No | No | Yes | Registrants must be 18 years of age for online registration. |
| South Carolina | 2009 | 13 | Yes^†^ | Exclude |  | Yes | Yes | No | Yes | Parental/guardian authorization is still sought at the time of procurement for those less than 18 years of age. In person registration is available through mobile laptops at various donation awareness events. |
| South Dakota | 2003 | 14 | No | n/a |  | No | Yes | No | Yes | Registrations made through a driver’s license or state identification card must be reaffirmed at renewal every five years. |
| Tennessee | 2008 | 13 | Yes^†^ | Include |  | Yes | Yes | No | Yes | Parental/guardian authorization is still sought at the time of procurement for those less than 18 years of age. |
| Texas | 2006 | None | Yes^†^ | Include & Exclude |  | Yes | Yes | No | Yes | Legislation passed in 2009 requires every Department of Public Safety office in Texas to ask whether or not someone wants to be an organ donor. |
| Utah | 2002 | None | Yes^†^ | Include & Exclude |  | Yes | Yes | Yes | Yes | While there is no age minimum to register, parental/guardian authorization is still sought at the time of procurement for those less than 18 years of age. |
| Vermont | 2007 | 18 | Yes^†^ | Exclude |  | Yes | Yes | No | No | Registration through the DMV is not available. |
| Virginia | 2003 | None | Yes^†^ | Exclude |  | Yes | Yes | No | Yes |  |
| Washington | 2003 | 15½ | Yes^†^ | Exclude |  | Yes | Yes | No | Yes |  |
| West Virginia | 1991 | None | No | n/a |  | Yes | Yes | No | Yes |  |
| Wisconsin | 2010^‡^ | 15½ | No | n/a |  | Yes | No | No | Yes | While registration does not expire, registrations must be affirmed while renewing a driver’s license or state identification card at the DMV. |
| Wyoming | 2003 | None | Yes^†^ | Include & Exclude |  | Yes | Yes | No | Yes | Registrants must be 16 years of age with signed parental/guardian authorization to register through the DMV. While there is no age minimum to register, parental/guardian authorization is still sought at the time of procurement for those less than 18 years of age. |

Note: DMV = Department of Motor Vehicles

^†^ Registrations made directly at the DMV do not provide the option to specify organs. Specification of organs to donate is available through online and/or paper registration.

^‡^ Authorization registry implemented to replace prior intent registry.

| **Appendix 3| Characteristics of Donor Registries Operating in Canadian Provinces** | | | | | | | | | | | |
| --- | --- | --- | --- | --- | --- | --- | --- | --- | --- | --- | --- |
| **Province** | **Implementation Date** | **Registration Choices** | **Minimum Age** | **Organ Specification** | |  | **Registration Modalities** | | | | **Additional Details** |
|  |  |  |  | **Can registrants specify which organs to donate?** | **Are specified organs to be included or excluded from donation?** |  | **Online** | **Paper** | **Telephone** | **In Person** |  |
| British Columbia | 1997 | Yes & No | None | Yes | Exclude |  | Yes | Yes | No | No | Registrants below the age of majority (19 years) must have their registration signed by a parent or guardian. |
| New Brunswick | 2007 | Yes & No | None | No | n/a |  | No | Yes | No | No | Registrants below the age of majority (18 years) must have their registration signed by a parent or guardian. Registration must be renewed every 3 years when the provincial health insurance card expires. |
| Nova Scotia | 1999 | Yes Only | 16 | Yes | Include |  | No | Yes | No | Yes | Registrants between the ages of 16-18 must have their registration signed by a parent or guardian until the age of majority (19 years). Registration must be renewed every 4 years when the provincial health insurance card expires. In person registration is available at awareness events. |
| Ontario | 1995 | Yes Only | 16 | Yes | Exclude |  | Yes | Yes | No | Yes | In person registration is available through ServiceOntario. It is also mandatory for ServiceOntario to ask any person not previously registered if they would like to be an organ donor during an in-person provincial health insurance card transaction. Online registration was implemented June 14^th^, 2011. |
| Quebec (Notary Public registry) | 2005 | Yes & No | 18 | No | n/a |  | No | No | No | Yes | In person registration is available through the Notary Public via a registered will or mandate for anticipated incapacity. |
| Quebec (RAMQ registry) | 2011 | Yes | 14 | No | n/a |  | No | Yes | Yes | Yes | Registrants below the age of 14 must have their registration signed by a parent or guardian. In person registration is available through Régie de l’assurance maladie du Quebec with renewal of the health insurance card. |
| Yukon | 2000 | Yes Only | None | Yes | Exclude |  | No | Yes | No | Yes | Registrants below the age of majority (18 years) must have their registration signed by a parent or guardian. Registration must be renewed every year when the provincial health insurance expires. |

| **Appendix 4| How the Donor Registry is Accessed and Utilized at the Time of Death in the United States** | | |
| --- | --- | --- |
| **State** | **How is the Registry Accessed by Staff** | **Authorized Personnel** |
| Alabama | Computer | OPOs, tissue banks and eye banks all have direct access. |
| Alaska | Computer | OPOs, tissue banks and eye banks all have direct access. |
| Arizona | Computer | OPOs, tissue banks and eye banks all have direct access. |
| Arkansas | Computer or Telephone | OPOs have direct access. Tissue banks and eye banks have access through the OPOs. |
| California | Computer | OPOs, tissue banks and eye banks all have direct access. |
| Colorado | Computer or Telephone | OPOs, tissue banks and eye banks and Stateline all have direct access. |
| Connecticut | Computer | OPOs, tissue banks and eye banks all have direct access. |
| Delaware | Computer | OPOs have direct access. Tissue banks and eye banks have access through the OPOs. |
| District of Columbia | Computer | OPOs have direct access. Tissue banks and eye banks have access through the OPOs. |
| Florida | Computer | OPOs, tissue banks and eye banks and Donate Life Florida administration all have direct access. |
| Georgia | Computer | OPOs and medical screener have direct access. Tissue banks and eye banks have access through the OPOs. |
| Hawaii | Computer or Telephone | Clinical, administrative and public education staff all have access. |
| Idaho | Computer or Telephone | OPOs, tissue banks and eye banks all have direct access. |
| Illinois | Telephone | OPOs, tissue banks, eye banks and coroners/medical examiners all have access. |
| Indiana | Computer or Telephone | OPOs, tissue banks and eye banks all have direct access. |
| Iowa | Computer | OPOs have direct access. Tissue banks and eye banks have access through the OPOs. |
| Kansas | Computer | OPOs, tissue banks and eye banks all have direct access. |
| Kentucky | Computer | OPOs, tissue banks and eye banks all have direct access. |
| Louisiana | Computer | OPOs and tissue banks have direct access. Eye banks have access through the OPOs. |
| Maine | Computer | OPOs, tissue banks and eye banks all have direct access. |
| Maryland | Computer | OPOs, eye banks and state registry staff all have access. |
| Massachusetts | Computer | OPOs, tissue banks and eye banks all have direct access. |
| Michigan | Computer | OPOs and DMV staff have direct access. Tissue and eye banks have access through the OPOs |
| Minnesota | Computer | OPOs, tissue banks and eye banks all have direct access. |
| Mississippi | Computer | OPOs and tissue banks have direct access. Eye banks have access through the OPOs. |
| Missouri | Computer | OPOs, tissue banks, eye banks and Department of Health and Senior Services staff all have direct access. |
| Montana | Computer | OPOs, tissue banks and eye banks have direct access. |
| Nebraska | Computer | OPOs, tissue banks and eye banks have direct access. |
| Nevada | Computer | OPOs, tissue banks and eye banks have direct access. |
| New Hampshire | Computer | OPOs, tissue banks and eye banks have direct access. |
| New Jersey | Computer | OPOs have direct access. Tissue banks and eye banks have access through OPOs. |
| New Mexico | Computer | OPOs, tissue banks and eye banks all have access. |
| New York | Computer | OPOs, tissue banks, eye banks and Department of Health staff all have direct access. |
| North Carolina | Computer | OPOs, tissue banks, eye banks and registry staff all have direct access. |
| North Dakota | Telephone | OPOs, tissue banks and eye banks all have access. |
| Ohio | Computer | OPOs, tissue banks and eye banks all have direct access. |
| Oklahoma | Computer | OPOs, tissue banks and eye banks all have direct access. |
| Oregon | Computer | OPOs, tissue banks and eye banks all have direct access. |
| Pennsylvania | Computer | OPOs have direct access. Tissue banks and eye banks have access through the OPOs. |
| Rhode Island | Computer | OPOs, tissue banks and eye banks all have direct access. |
| South Carolina | Computer | OPOs, tissue banks, eye banks and registry staff all have direct access. |
| South Dakota | Telephone | OPOs, tissue banks and eye banks all have access. |
| Tennessee | Computer | OPOs have direct access. Tissue banks and eye banks have access through OPOs. |
| Texas | Computer | OPOs, tissue banks and eye banks all have direct access. |
| Utah | Computer or Telephone | OPOs, tissue banks and eye banks all have direct access. |
| Vermont | Computer | OPOs have access. |
| Virginia | Computer | OPOs, tissue banks and eye banks all have direct access. |
| Washington | Computer | OPOs, tissue banks and eye banks all have direct access. |
| West Virginia | Computer | OPOs have access. |
| Wisconsin | Computer | OPOs, tissue banks and eye banks all have access. |
| Wyoming | Computer or Telephone | OPOs, tissue banks, eye banks and Statline all have direct access. |

Note: All state registries are legally-binding authorization registries. OPO = organ procurement organization, DMV = department of motor vehicles

| **Appendix 5| How the Donor Registry is Accessed and Utilized at the Time of Death in Canada** | | | |
| --- | --- | --- | --- |
| **Province** | **Intent or Legal Authorization** | **How is the Registry Accessed by Staff** | **Authorized Personnel** |
| British Columbia | Legal Authorization | Computer | Provincial Health Services Authority/BC Transplant staff and British Columbia health care providers. |
| New Brunswick | Intent | Computer | Organ donation resource nurses (4 in total) and a healthcare consultant (1 in total). |
| Nova Scotia | Legal Authorization | Computer | Transplant coordinators and regional tissue bank staff. |
| Ontario | Legal Authorization | Computer & Telephone^†^ | ServiceOntario 24/7 help desk staff and Trillium Gift of Life staff. |
| Quebec^‡^ | Legal Authorization | Computer | Quebec-Transplant staff (22 personnel in total) and Hema-Quebec staff (7 personnel in total) have access to both registries. Hospital staff at two hospitals (6 personnel in total) only have access to the Notary Public’s registry, not the RAMQ registry. |
| Yukon | Intent | Computer | Ministry of Health department staff, and hospital personnel (including Emergency and Medicine staff). |

^†^ On a donor referral, a transplant coordinator phones the ServiceOntario 24/7 help desk and provides the deceased’s full name, health number, gender and date of birth. The help desk agent then

checks the Registered Persons Database to search for a match and shares the information verbally by phone and electronically in an encrypted email.

^‡^ Both Quebec registries must be checked after a donor referral. If an individual is listed on only one registry, their intentions are noted and proceeded with accordingly. If the individual answered ‘yes’ to both registries, authorization is considered given. If there is conflicting registrants, the date of the most recent registration determines the course of action. In all cases, an attempt must be made to also check if the person signed his/her health insurance card, since as of 2010 the organ donation sticker has included a date, which may differ from the registry/registries and therefore may have an impact on the individual’s wishes. More information can be found in the Quebec Civil Code.

| **Appendix 6 | Number and Proportions of Registrants for United States** | | | |
| --- | --- | --- | --- |
| **State** | **Total Adult Population** | **Total Registrants (Affirmative)** | **Proportion of Population Registered (Affirmative) (%)** |
| Alabama | 3,579,844 | 1,948,496 | 54.43 |
| Alaska | 514,927 | 402,694 | 78.20 |
| Arizona | 4,863,759 | 1,696,681 | 34.88 |
| Arkansas | 2,179,482 | 1,249,570 | 57.33 |
| California | 27,525,982 | 8,176,218 | 29.70 |
| Colorado | 3,796,985 | 2,450,000^†^ | 64.52 |
| Connecticut | 2,710,303 | 1,053,680 | 38.88 |
| Delaware | 678,129 | 308,704 | 45.52 |
| District of Columbia | 485,621 | 191,904 | 39.52 |
| Florida | 14,480,196 | 5,771,017 | 39.85 |
| Georgia | 7,245,419 | 3,897,886 | 53.80 |
| Hawaii | 1,004,817 | 489,951 | 48.76 |
| Idaho | 1,126,611 | 666,073 | 59.12 |
| Illinois | 9,733,032 | 5,628,360 | 57.83 |
| Indiana | 4,833,748 | 3,255,073 | 67.34 |
| Iowa | 2,294,701 | 1,225,000^†^ | 53.38 |
| Kansas | 2,113,796 | 650,066 | 30.75 |
| Kentucky | 3,299,790 | 1,069,121 | 32.40 |
| Louisiana | 3,368,690 | 1,942,604 | 57.67 |
| Maine | 1,047,125 | 515,726 | 49.25 |
| Maryland | 4,347,543 | 2,151,981 | 49.50 |
| Massachusetts | 5,160,585 | 2,344,566 | 45.43 |
| Michigan | 7,619,835 | 2,140,663 | 28.09 |
| Minnesota | 4,005,417 | 2,386,740 | 59.59 |
| Mississippi | 2,184,254 | 430,664 | 19.72 |
| Missouri | 4,556,242 | 2,829,786 | 62.11 |
| Montana | 755,161 | 586,604 | 77.68 |
| Nebraska | 1,344,978 | 654,393 | 48.65 |
| Nevada | 1,962,052 | 803,000^†^ | 40.93 |
| New Hampshire | 1,035,504 | 207,853 | 20.07 |
| New Jersey | 6,661,891 | 2,187,212 | 32.83 |
| New Mexico | 1,499,433 | 935,000^†^ | 62.36 |
| New York | 15,117,370 | 2,374,163 | 15.70 |
| North Carolina | 7,102,917 | 4,064,489 | 57.22 |
| North Dakota | 502,873 | 324,912 | 64.61 |
| Ohio | 8,828,304 | 5,013,384 | 56.79 |
| Oklahoma | 2,768,201 | 1,767,276 | 63.84 |
| Oregon | 2,952,846 | 2,121,620 | 71.85 |
| Pennsylvania | 9,829,635 | 4,359,681 | 14.35 |
| Rhode Island | 826,384 | 364,130 | 44.06 |
| South Carolina | 3,480,510 | 649,055 | 18.65 |
| South Dakota | 612,767 | 336,699 | 54.95 |
| Tennessee | 4,803,002 | 1,518,595 | 31.62 |
| Texas | 17,886,333 | 1,459,595 | 8.16 |
| Utah | 1,915,748 | 1,265,623 | 66.06 |
| Vermont | 495,485 | 1,631 | 0.33 |
| Virginia | 6,035,408 | 3,592,543 | 59.52 |
| Washington | 5,094,603 | 3,759,850 | 73.80 |
| West Virginia | 1,433,328 | 495,903 | 34.60 |
| Wisconsin | 4,344,524 | 2,446,600 | 56.31 |
| Wyoming | 412,245 | 255,000^†^ | 61.86 |

Note: Adult population is defined as those 18 years of age and older, and was calculated from U.S. Census Bureau. Exact proportions will vary slightly for states with no minimum age and for those with age minimums lower than 18. Please see Appendix 2 for each state’s minimum age requirements. All values are current as of 3/31/11,

^†^ Estimated values

| **Appendix 7 | Number and Proportions of Registrants for Canada** | | | | | | |
| --- | --- | --- | --- | --- | --- | --- |
| **Province** | **Total Adult Population** | **Total Registrants** | **Total Affirmative Registrants** | **Total Objecting Registrants** | **Proportion of Population Registered (%)** | **Proportion of Population Registered as Affirmative (%)** |
| British Columbia | 3,844,600 | 782,115^†^ | 323,538 | 3502 | 20.34 | 8.42 |
| New Brunswick | 638,300 | 498,159 | 267,828 | 230,331 | 78.04 | 41.96 |
| Nova Scotia | 802,700 | 518,990 | 518,990 | - | 64.66 | 64.66 |
| Ontario | 11,005,000 | 1,965,992 | 1,965,992 | - | 17.86 | 17.86 |
| Quebec | 6,670,700 | 650,000 | 633,500^‡^ | 16,500 | 9.74 | 9.50 |
| Yukon | 28,600 | 4,548 | 4,548 | - | 15.90 | 15.90 |

Note: Adult population is defined as those 15 years of age and older, and was calculated from Statistics Canada. Exact proportions will vary slightly for nations with no minimum age and for those with age minimums higher than 15. Please see Appendix 3 for each province’s minimum age requirements. Values are current as of the following dates: November 2010 (NB), December 2010 (NS,ON), January 2011 (BC, QC-Notary), March 2011 (YK) & June 2011 (QC-RAMQ)

^†^ British Columbia has 455,075 registrations that are considered invalid or have errors

^‡^ Includes registrations from Notary registry (533,500) and the RAMQ registry (estimated 100,000)
